# Event Analysis for Automated Estimation of Absent and Persistent Medication Alerts: Novel Methodology

**DOI:** 10.2196/54428

**Published:** 2024-06-04

**Authors:** Janina A Bittmann, Camilo Scherkl, Andreas D Meid, Walter E Haefeli, Hanna M Seidling

**Affiliations:** 1 Internal Medicine IX: Department of Clinical Pharmacology and Pharmacoepidemiology, Cooperation Unit Clinical Pharmacy, Heidelberg University, Medical Faculty Heidelberg/Heidelberg University Hospital, Heidelberg, Germany; 2 Internal Medicine IX: Department of Clinical Pharmacology and Pharmacoepidemiology, Heidelberg University, Medical Faculty Heidelberg/Heidelberg University Hospital, Heidelberg, Germany

**Keywords:** clinical decision support system, CDSS, medication alert system, alerting, alert acceptance, event analysis

## Abstract

**Background:**

Event analysis is a promising approach to estimate the acceptance of medication alerts issued by computerized physician order entry (CPOE) systems with an integrated clinical decision support system (CDSS), particularly when alerts cannot be interactively confirmed in the CPOE-CDSS due to its system architecture. Medication documentation is then reviewed for documented evidence of alert acceptance, which can be a time-consuming process, especially when performed manually.

**Objective:**

We present a new automated event analysis approach, which was applied to a large data set generated in a CPOE-CDSS with passive, noninterruptive alerts.

**Methods:**

Medication and alert data generated over 3.5 months within the CPOE-CDSS at Heidelberg University Hospital were divided into 24-hour time intervals in which the alert display was correlated with associated prescription changes. Alerts were considered “persistent” if they were displayed in every consecutive 24-hour time interval due to a respective active prescription until patient discharge and were considered “absent” if they were no longer displayed during continuous prescriptions in the subsequent interval.

**Results:**

Overall, 1670 patient cases with 11,428 alerts were analyzed. Alerts were displayed for a median of 3 (IQR 1-7) consecutive 24-hour time intervals, with the shortest alerts displayed for drug-allergy interactions and the longest alerts displayed for potentially inappropriate medication for the elderly (PIM). Among the total 11,428 alerts, 56.1% (n=6413) became absent, most commonly among alerts for drug-drug interactions (1915/2366, 80.9%) and least commonly among PIM alerts (199/499, 39.9%).

**Conclusions:**

This new approach to estimate alert acceptance based on event analysis can be flexibly adapted to the automated evaluation of passive, noninterruptive alerts. This enables large data sets of longitudinal patient cases to be processed, allows for the derivation of the ratios of persistent and absent alerts, and facilitates the comparison and prospective monitoring of these alerts.

## Introduction

Computerized physician order entry (CPOE) systems with integrated clinical decision support systems (CDSS) can reduce medication errors by highlighting critical medication constellations [1]. To realize their full potential, medication alerts must be recognized and followed by users. Hence, measuring “alert acceptance” is a key prerequisite for evaluating the effectiveness of a CDSS.

In principle, two methods can estimate alert acceptance: (1) in-dialog analysis where users interactively click to accept or override displayed alerts; and (2) event analysis where the medication chart and associated documentation are reviewed for evidence of alert acceptance through further actions (“events”) responsive to the alert (eg, discontinued medication orders), which often requires extensive manual screening [2]. Most studies addressing alert acceptance used in-dialog analyses because the display of alerts, especially in English-speaking countries, is part of the technical architecture of the CPOE-CDSS [3]. There is limited evidence on how to perform event analyses because it is uncertain whether the prescribing behavior is influenced by alerts or other clinical therapeutic circumstances (eg, scheduled end of treatment) [2]. Moreover, the manual screening of the medication documentation is a time-consuming process [4], especially when administrative processes such as changing wards and the simultaneous transfer of physicians’ responsibility for the medication are considered in the alert presentation.

As CDSS installations presenting passive, noninterruptive alerts become increasingly popular in European countries [5,6], the need for developing and validating techniques for automatic event analyses is increasing. This is particularly important when considering all alerts throughout the inpatient stay.

We present a new approach to perform an automated event analysis, which was applied to a large data set of medication alerts.

## Methods

### Ethical Considerations

Study approval was granted by the responsible Ethics Committee of the Medical Faculty of Heidelberg University (S-467/2020) and by the local data protection officer for the data protection concept. Human subjects were not directly involved; all data were pseudonymized and could neither be linked to individual patients nor to prescribers.

### Setting

**Figure 1.**
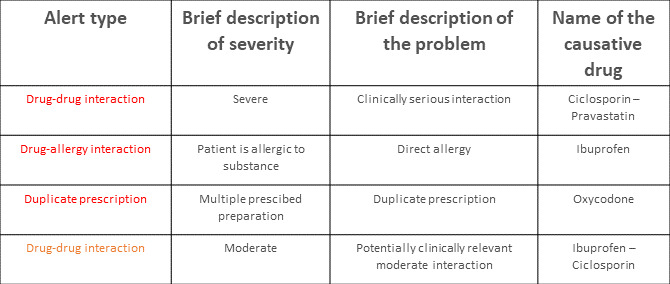
Schematic display of an exemplary alert window listing all alerts for a patient in a table. Each alert is presented in a separate line, sorted by severity, with the most severe alerts listed first. The first column displays the alert type in a color-coded scheme (black=contraindicated, red=severe, orange=moderate), followed by an explanation for the severity, a brief description of the problem, and the name of the causative drug.

We analyzed the prescription and alert data issued over 3.5 months during routine care at Heidelberg University Hospital (a 2500-bed tertiary care hospital) within the local CPOE (*i.s.h.med Smart Medication,* Oracle Cerner, North Kansas City, USA) with an integrated CDSS (AiD*Klinik,* Dosing GmbH*,* Heidelberg, Germany). To view the presented passive and noninterruptive alerts, users actively navigate from their prescription screen to a separate window that opens upon request. In this window, all alerts are displayed in a single table sorted by severity and presented with a brief summary (Figure 1). Users are required to click on each alert to access more detailed information. The system does not recognize whether an alert has been viewed. Additionally, users are not obliged to directly flag alerts as accepted or overwritten. Therefore, these data are not available in our CPOE-CDSS. Implemented alert types comprised checking for drug-drug interactions (DDIs), drug-allergy interactions (DAIs), duplicate prescriptions (DPs), advanced dosing recommendations for potentially inappropriate medication for the elderly (aged ≥65 years, PIM), or prescriptions exceeding the maximum recommended daily dose (PE-MDDs).

### Data Collection

The relevant parameters extracted from the CPOE-CDSS were information on prescriptions, issued alerts, administrative patient data, and setting data. Prescription schedules with regimen changes were documented as separate entries so that prescriptions potentially resulting from previous prescriptions (eg, because of dose reduction or conversion of fixed to as-needed prescriptions) could be linked retroactively. Follow-up prescriptions were defined as prescriptions of the same drug and administration route when the previous prescription ended and the subsequent one started within 10 minutes.

### Alert Management

In this CDSS, prescriber review of alerts may result in alerts disappearing due to prescription changes and adaptations or in alerts being continuously displayed for unchanged prescriptions.

In this methodology, alerts are defined as “absent” when they disappear during continuous prescriptions for which underlying risk constellations no longer exist (eg, dose reduction of an overdosed prescription but the prescription itself remains continuous). In contrast, alerts consistently displayed until patient transfer, discharge, or end of the prescription are categorized as “persistent” (eg, the prescription remains valid even though the prescribed active ingredient is alerted by a DAI).

### Data Analysis

**Figure 2.**
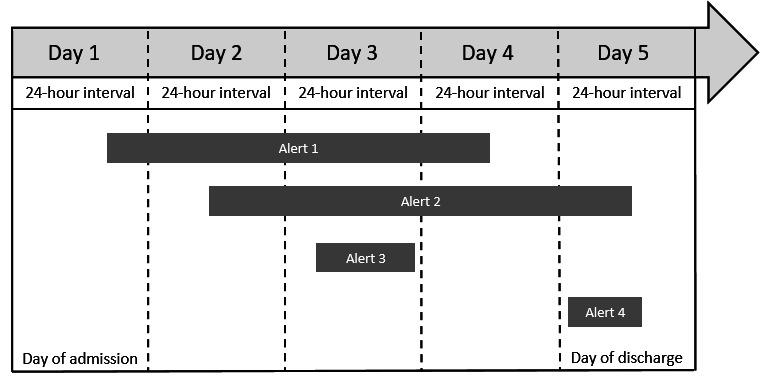
Proposed methodology for identification of absent alerts, exemplified for a 5-day inpatient stay. Each midnight (dotted lines), all alerts displayed within the last 24-hour time interval were identified. Alert 1 is displayed between day 1 (admission) and day 4; the display duration of alerts (DDoA) is 4 days. Alert 2 is displayed from day 2 until discharge; the DDoA is 4 days. Alert 3 is displayed only on day 3; the DDoA is 1 day. Alert 4 is displayed for the first time on the day of discharge and remained until discharge; the DDoA is 1 day. Alert 4 was excluded from the analysis because, due to the discharge, there is no subsequent (sixth) 24-hour time interval with which the fifth interval could have been compared to evaluate the alert display. Each alert could be identified because of a unique alert ID code. Using this identification concept, alert IDs detected within a 24-hour time interval could be compared to alerts detected in the previous 24-hour time interval. Therefore, it was possible to automatically classify which alerts were (1) newly displayed (no matching ID in the previous interval: Alert 1, Day 1), (2) displayed for more than 24 hours (matching ID in consecutive intervals; Alert 1, Days 2-4), or (3) absent (no matching ID in the current 24-hour time interval: Alert 1, Day 4).

To automatically identify absent alerts, the medication and corresponding alert data were divided into 24-hour time intervals. Since reproducible time stamps were lacking in the database, selection of this conservative interval allowed the retroactive linkage of alert display and associated changes in the corresponding prescription. Therefore, two consecutive time intervals were compared according to whether or not the alerts were continuously displayed. Alerts were excluded if (1) they were first displayed on the discharge day (Figure 2, Alert 4); (2) required user interaction (eg, answering questions to decide whether conditions for alerts are present); or (3) were triggered by one-time prescriptions, in which case the alert response cannot be assessed the next day (Supplementary Methods section A and Figure S1 in Multimedia Appendix 1). The display duration of alerts (DDoA) was calculated from the time interval between the first and last alert display (Figure 2). Further details on the development of the 24-hour time intervals can be found in Supplementary Methods section B in Multimedia Appendix 1; examples of data analysis of different alert types are provided in Tables S1-S3 and Figure S2 of Multimedia Appendix 1.

Based on the exploratory rates of absent alerts, basic descriptive statistics were applied. The *χ*^2^ test was performed to evaluate whether absent alerts differed stratified by alert types considering a two-tailed *P* value <.05 as significant (IBM SPSS Statistics version 25, Ehningen, Germany). The R packages *gpmodels* [7], *survival*, and *ggplot2* (version 4.1.2, R Foundation for Statistical Computing, Vienna, Austria) were used for data analysis and visualization.

## Results

### Alert Display and Composition

We considered the data of 1670 patient cases (Figure S3 in Multimedia Appendix 1) with a median hospital stay of 7 days (IQR 4-13). During this time, 13,979 alerts were displayed. Because 2284 alerts (16.3%) were triggered by one-time prescriptions and 267 alerts (1.9%) were first displayed on the discharge day, the remaining 11,428 alerts (81.8%) formed the basis for analysis. The alert types triggering the alerts are shown in Table 1.

The median DDoA was 3 days (IQR 1-7) and varied by alert type, with alerts for DAIs showing the shortest DDoA (Table 1).

**Table 1.** Alert types triggering the alerts, corresponding rates of absence, and display duration of the alerts.

Alert type	Triggered alerts, n (%)^a^	Absent alerts, n (%)^b^	Display duration of alerts (days), median (IQR; range)
Alerts for duplicate prescriptions	7643 (66.9)	3674 (48.1)	3 (1-8; 1-31)
Alerts for drug-drug interactions	2366 (20.7)	1915 (80.9)	2 (1-5; 1-31)
Alerts for drug-allergy interactions	517 (4.5)	416 (80.5)	1 (1-2; 1-24)
Alerts for potentially inappropriate medication for the elderly	499 (4.4)	199 (39.9)	4 (2-8; 1-31)
Alerts for prescriptions exceeding the maximum recommended daily dose	403 (3.5)	209 (51.9)	3 (1-6; 1-30)
Total number of alerts	11,428	6413	3 (1-7; 1-31)

a Percentages are based on the total number of analyzed alerts (N=11,428).

b Percentages are based on the number of analyzed alerts for each alert type.

### Absent and Persistent Alerts

From all 11,428 analyzed alerts, 43.9% (n=5015) persisted and 56.1% (n=6413) were absent, with alerts for DDIs showing the highest rate of absence (80.9%) and PIMs the lowest (39.9%) (Table 1).

The proportions of absent alerts differed significantly between the individual alert types (*P*_χ^2^_<.001), except for DDI alerts compared to DAI alerts (*P*_χ^2^_=.80) and for DP alerts compared to alerts for PE-MDDs (*P*_χ^2^_=.14). The proportion of absent alerts in relation to the DDoA was the highest for DAI alerts and the lowest for alerts for PIMs in the first 24 hours after admission (Figures S4-S5 in Multimedia Appendix 1).

## Discussion

### Principal Findings

A new methodological approach for routine care data was applied performing an automated event analysis that is transferable to other CPOE-CDSS with passive, noninterruptive alerts. In previous studies using event analyses, alert acceptance rates were identified at the drug administration level [8], prescription level [9], or at both levels [10]. There is general consensus that alert acceptance rates vary widely depending on the measuring method and study setting, resulting in different and incomparable rates [11]. Since in-dialog analysis is often not possible in a European CPOE-CDSS, this new methodology adapted to the technical structures of such a CPOE-CDSS is needed.

A key strength of the proposed method is that it variably adjusts the time intervals and consequently the lookback windows underlying the method’s programming. Thus, temporary changes in prescriptions within the determined time interval (here 24 hours) are considered persistent alerts; however, this CPOE-CDSS interrupts the alert display in certain cases, such as when patients change wards and responsibility for medication is handed over to another physician. This transfer results in automatic prescription pauses that are actively suspended by physicians, technically leading to the redisplay of alerts. Without the definition of this time interval, these pauses would incorrectly increase the overall number of alerts when reappearing and the rate of absent alerts as they disappear for a few hours during valid prescriptions. Hence, this method considers administrative processes of the daily clinical routine and guarantees that only alerts of real prescription changes are evaluated. For retrospectively matching the time-dependent correlations of the alerts over time and in the clinical routine, it is essential to consider alerts throughout the inpatient stay and our proposed method meets this need.

However, according to the technical architecture of this CPOE-CDSS, there is no obvious link between reviewing alerts and possible resulting changes in prescription data. Therefore, various assumptions had to be made for this data evaluation. Alerts were categorized as either persistent or absent based on the assumptions that alerts were regularly checked and that alerts disappeared because underlying risk constellations no longer existed due to previously displayed alerts. This general assumption may overestimate the rate of actual alert acceptance, as a prescribed medication could be switched based on patient conditions (eg, adverse events, intolerance) or treatment schedules. As it remains unclear whether the change in drug prescriptions was caused by the alert display or due to other variables and because no control group was available due to the retrospective design, caution is required when interpreting absolute numbers and comparing the proportion of absent alerts to previously published acceptance rates. In the future, this retrospective method will need to be prospectively evaluated including validity measurements by comparing the results of this automated approach with those derived from manual screening. Another limitation is that this study was conducted in a single center with a CPOE-CDSS that is highly specific and strongly adapted to workflows and care processes at our institution. This analysis only considered alerts at the prescribing level and did not measure whether the respective drugs were indeed administered. For instance, many of the alerts for DPs were triggered by drugs that were prescribed as as-needed prescriptions. Hence, these DPs tended to indicate a variety of treatment options rather than actually being administered together. This might have contributed to the reduced occurrence of the absence of alerts for DPs on a prescribing level compared to other alerts. However, in our CPOE-CDSS, it is unalterably stipulated that medication alerts are implemented in a passive and noninterruptive way. While it may be challenging to transfer this complex method to systems with differing data infrastructures, to our knowledge, this is the first automated method for processing persistent and absent medication alerts in a system with passive, noninterruptive alerts. Additionally, since this method was programmed in a modular way, it seems feasible to transfer and adapt it to other settings.

### Conclusions

A methodology was applied to an automatic event analysis in a CPOE-CDSS with passive, noninterruptive alerting. This enables the processing of large data sets of longitudinal periods of inpatient stays and can be used to automatically derive the percentage of absent alerts. Once implemented, this analysis can be repeated at any time and one could even imagine that real-time monitoring of persistent alerts in daily clinical routines could be set up using these data for future optimization of the CPOE-CDSS.

## Supplementary material

10.2196/54428 Multimedia Appendix 1 Description of one-time prescription and illustration of its impact on the alert display (Figure S1). Description of the development of 24-hour time intervals, with exemplary data sets for fixed variables (Table S1), time-dependent variables (Table S2), and an exemplary time frame of processed longitudinal alert data (Table S3). Data analysis examples for different alert types (Figure S2). CONSORT (Consolidated Standards of Reporting Trials) diagram for included patient cases (Figure S3). Proportions of absent (Figure S4) and persistent (Figure S5) alerts stratified by the alert type.

## Abbreviations

CDSS: clinical decision support system
CPOE: computerized physician order entry
DAI: drug-allergy interaction
DDI: drug-drug interaction
DDoA: display duration of alert
DP: duplicate prescription
PE-MDD: prescription exceeding the maximum recommended daily dose
PIM: potentially inappropriate medication for the elderly
