# Supplementary material for: Event Analysis for Automated Estimation of Absent and Persistent Medication Alerts: Novel Methodology
Source: JMIR Med Inform. 2024 Jun 4;12:e54428. doi: 10.2196/54428 (PMC11185280; doi:10.2196/54428)
Supplement: Multimedia Appendix 1 [file medinform-v12-e54428-s001.docx]

**Multimedia Appendix 1**

**SUPPLEMENTARY METHODS SECTION A:** One-time prescriptions

One-time prescriptions are defined as prescriptions that were only prescribed for one event, regardless of whether they were prescribed by physicians or documented as an urgent administration by nursing. Consequently, alerts resulting from one-time prescriptions were only displayed for the day of the event. Thus, alerts resulting solely from one-time prescriptions were not included in this analysis as the evaluation of prescriber’s reaction thereto is not useful. Exceptions had to be defined for the case that one-time prescriptions exist simultaneously with ongoing continuous prescriptions of the same active ingredient triggering the same alert.

The approach may be illustrated using the example of a patient with acute pain and analgesics: According to the WHO pain level scheme, oxycodone extended-release is prescribed as a continuous prescription and for breakthrough pain, immediate-release oxycodone is acutely administered by nursing. Both oxycodone prescriptions trigger alerts for DDIs with comedication like vinflunine (Figure S1). The alert for DDI of the continuous prescriptions is displayed for the entire duration of the simultaneously prescribed substances (Figure S1, i)), whereas for an urgent administered oxycodone by nursing staff it is only displayed for the same day (Figure S1, ii)).

Assuming that physicians cannot respond to alerts triggered by one-time prescriptions as this prescription has already stopped by definition, and as the used methodology always requires a second consecutive timepoint on the next day to compare alert display, these cases were excluded for further analysis. Thus, alerts which are solely based on one-time prescriptions were excluded unless the active ingredient was already prescribed at the same time.


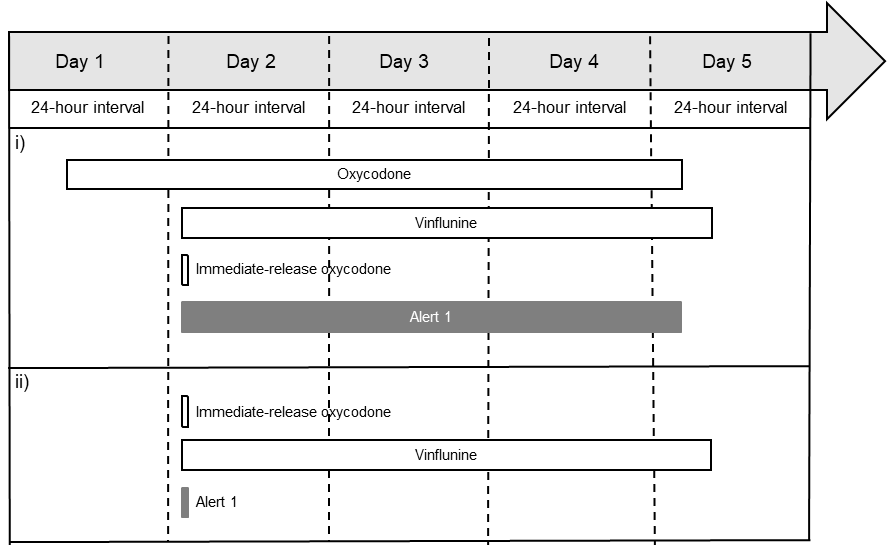


**Figure S1: Illustration of one-time prescription and impact of alert display.** Oxycodone and vinflunine prescriptions trigger Alert 1. i) shows two continuous prescriptions (oxycodone and vinflunine) as well as one one-time prescription of immediate-release oxycodone. The alert is mainly triggered by the two continuous prescriptions, an appropriate user reaction to the alert can be assessed as consecutive intervals for the alert are available. ii) shows only the one-time prescription of immediate-release oxycodone and one continuous prescription of vinflunine. This alert triggered by the one-time prescription was excluded from analysis as it is not displayed in the consecutive interval and its ID is consequently missing therein. In this example, the user’s response cannot be evaluated because the one-time prescription, by definition, ends on the same day and cannot trigger an alert the next day.

**SUPPLEMENTARY METHODS SECTION B:** Development of 24-hour time intervals

To minimize excessive alert data (repeating entries of same alerts triggered by multiple prescription checks per day), only the first chronologically unique alert of each prescription for each day was identified by timestamps. Then, to automatically generate the time intervals and identifying the display duration of alerts (DDoA), two datasets with fixed variables (Table S1) and time-dependent variables (Table S2) were generated with patient cases as observational unit. Fixed variables are defined as permanent variables (i.e., demographics, admission dates, and discharge dates), while time-dependent variables can change their value over time (i.e., alert display).

**Table S1: Exemplary dataset of fixed variables.** Fixed variables that were constant over the study period consisted of patient’s pseudonym, admission date, and discharge date. Admission and discharge dates are crucial for the development of the time intervals in 24-hour steps beginning from the day of admission until the day of discharge.

| pseudonym | admission | Discharge |
| --- | --- | --- |
| 1234 | 2021-01-01 | 2021-01-03 |
| 1345 | 2021-01-01 | 2021-01-03 |

**Table S2: Exemplary dataset of time-dependent variables.** Time-dependent variables show the dates of the alerts displayed (ALERT TIMESTAMP) and define the displayed alerts (ALERT ID) as well as the alert type (ALERT TYPE). This organized breakdown of alert data allows the chronological tracking of each generated alert during the study period.

**Abbreviations:** DDI: Alerts for drug-drug interactions, ID: Identification code, PIM: Alerts for potentially inappropriate medication for patients aged ≥ 65 years.

| pseudonym | Alert Timestamp | Alert ID | Alert type |
| --- | --- | --- | --- |
| 1234 | 2021-01-01 | DDI_12345 | DDI |
| 1234 | 2021-01-02 | DDI_12345 | DDI |
| 1234 | 2021-01-03 | DDI_12345 | DDI |
| 1345 | 2021-01-01 | PIM_02564 | PIM |
| 1345 | 2021-01-02 | PIM_02564 | PIM |

After using the function *time_frame* from the R package *gpmodels* and setting the time intervals to 24 hours, a new longitudinal data frame (Table S3) was generated presenting a list of all alerts that occurred during the study period.

**Table S3: Exemplary timeframe of processed longitudinal alert data.** The application of the 24-hour intervals resulted in one row per alert ID and per day between admission and discharge. The factor 1 denotes the alert display within the respective 24-hour interval and the factor 0 denotes the absence of the alert.

**Abbreviations:** 0: Alert is not displayed in the respective 24-hour time interval, 1: Alert is displayed in the respective 24-hour time interval, DDI: Alerts for drug-drug interactions, h: hour, PIM: Alerts for potentially inappropriate medication for patients aged ≥ 65 years, TIME: 24-hour time intervals between admission and discharge per patient case.

| pseudonym | admission | Discharge | Time [h] | DDI_12345 | PIM_02564 |
| --- | --- | --- | --- | --- | --- |
| 1234 | 2021-01-01 | 2021-01-03 | 0 | 1 | 0 |
| 1234 | 2021-01-01 | 2021-01-03 | 24 | 1 | 0 |
| 1234 | 2021-01-01 | 2021-01-03 | 48 | 1 | 0 |
| 1345 | 2021-01-01 | 2021-01-03 | 0 | 0 | 1 |
| 1345 | 2021-01-01 | 2021-01-03 | 24 | 0 | 1 |
| 1345 | 2021-01-01 | 2021-01-03 | 48 | 0 | 0 |

The change of the factors from 1 or 0 describes the beginning or end of the display of an alert. Comparison with the corresponding prescription data (i.e., with start, end dates, or a pause in prescribing) allows identification of absent and persistent alerts.

**SUPPLEMENTARY METHODS SECTION C:** Data analysis of the different alert types

| **i) Exemplary alert management for DDIs or DPs**  **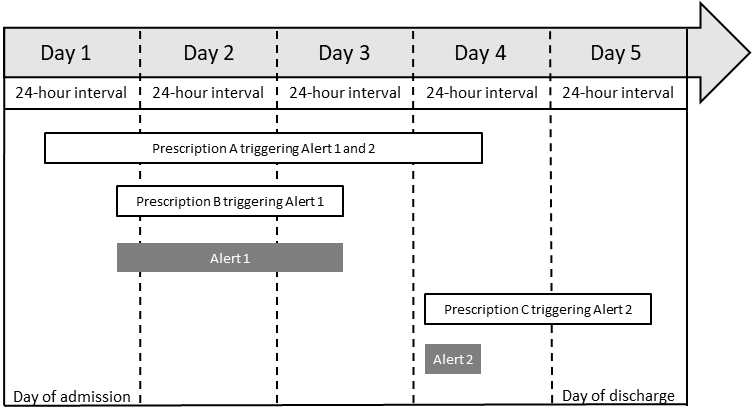** | **ii) Exemplary alert management for DAIs, PIMs, and prescriptions exceeding the MDD**  **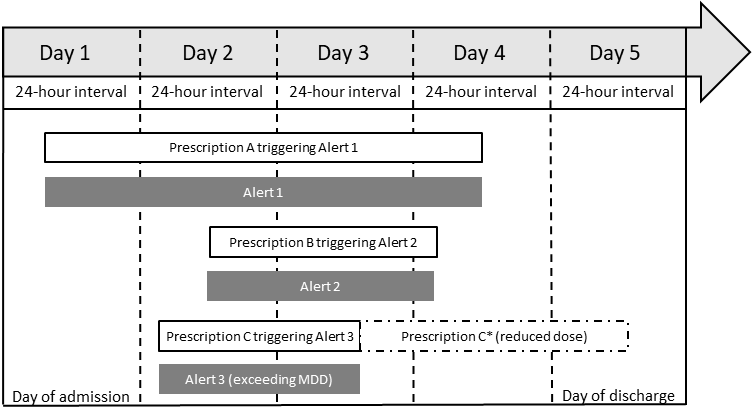** |
| --- | --- |

**Figure S2: Exemplary alert management for alert types with at least two prescriptions triggering an alert i) (e.g., alerts for DDIs or DPs) and for alert types with one prescription triggering an alert ii) (e.g., alerts for DAIs, PIMs, and prescriptions exceeding the MDD).** i) The simultaneous prescription of prescription A and B or A and C triggers an alert for the corresponding days in each case (Alert 1 and 2, respectively). ii) Prescription A or B triggers Alert 1 or 2 when a (cross-)allergy for the corresponding active ingredient is documented or when an alert for PIM is triggered. Prescription C triggers Alert 3 when its dosing regimen exceeds the MDD. The alert disappears in the subsequent 24-hour interval after the dosing regimen is reduced so that the MDD is no longer exceeded.

**Abbreviations:** DAIs: Alerts for drug-allergy interactions, DDIs: Alerts for drug-drug interactions, DPs: Duplicate prescriptions, MDD: Prescriptions exceeding the maximum recommended daily dose, PIMs: Alerts for potentially inappropriate medication for patients aged ≥ 65 years.

Patient cases during study period

(n = 2,787)

Cases meeting inclusion criteria

(n = 2,782)

Included cases

(n = 1,670)

**Exclusion criteria**

Cases without alerts (n = 47)

Technical mistakes (n = 536)

Alerts requiring user interaction (n = 529)

**Inclusion criteria not met**

Cases without prescriptions triggering an alert (n = 4)

Incomplete baseline characteristics (n = 1)

**Figure S3**: **CONSORT diagram for excluded and included patient cases in the study period.** **Abbreviation:** n: number.


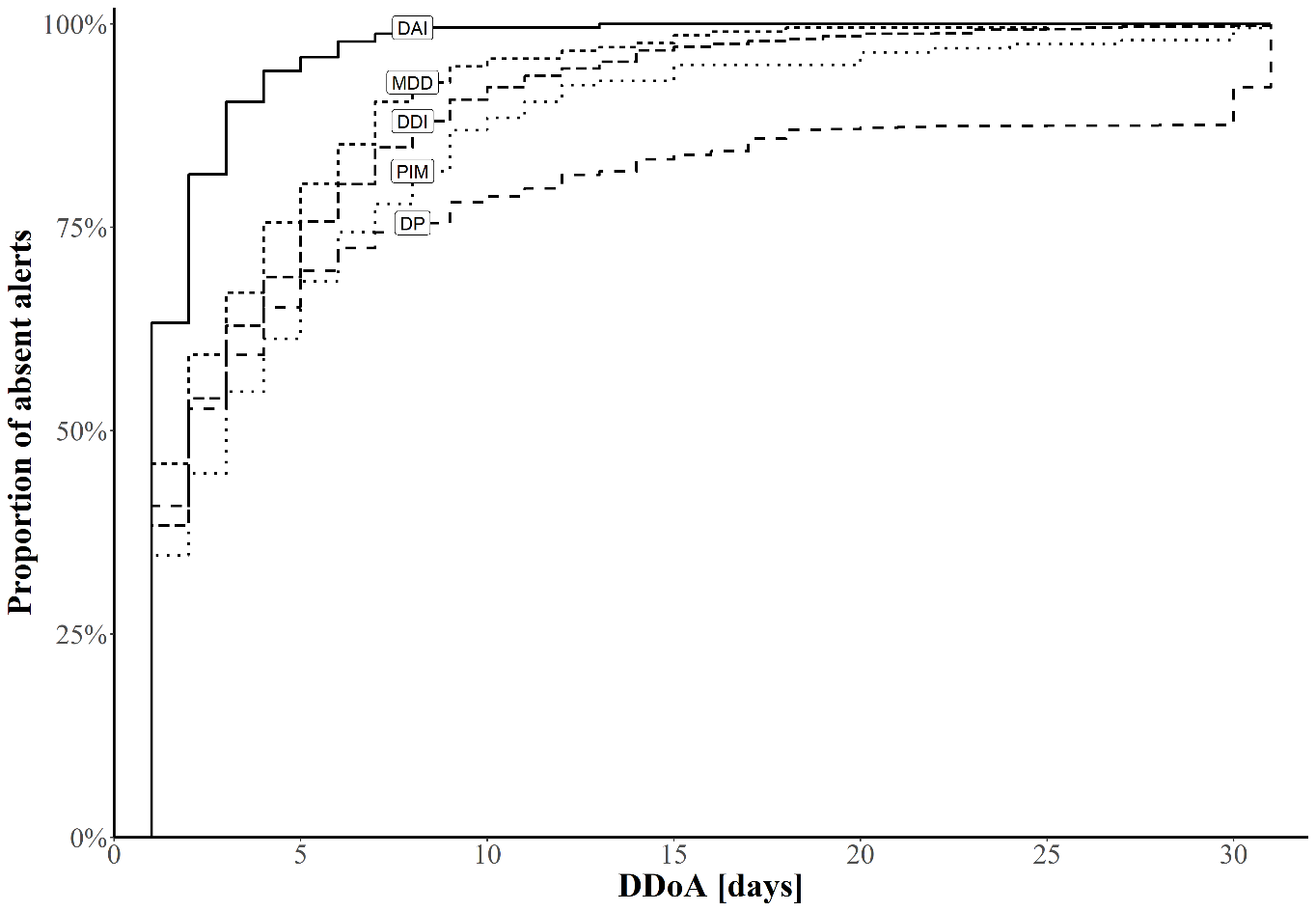


***Figure S4:*** ***Proportion of absent alerts over the displayed period stratified by the alert type.*** *Within each 24-hour time interval (one day), alerts are absent so that the curves plotted by alert type at these times stepwise approach the 100 % mark of absent alerts. Only absent alerts were considered for plotting.*

***Abbreviations:*** *DAI: Alerts for drug-allergy interactions, DDI: Alerts for drug-drug interactions, DDoA: Display duration of alerts, DP: Alerts for duplicate prescriptions, MDD: Alerts for prescriptions exceeding the maximum recommended daily dose, PIM: Alerts for potentially inappropriate medication for patients aged ≥ 65 years.*


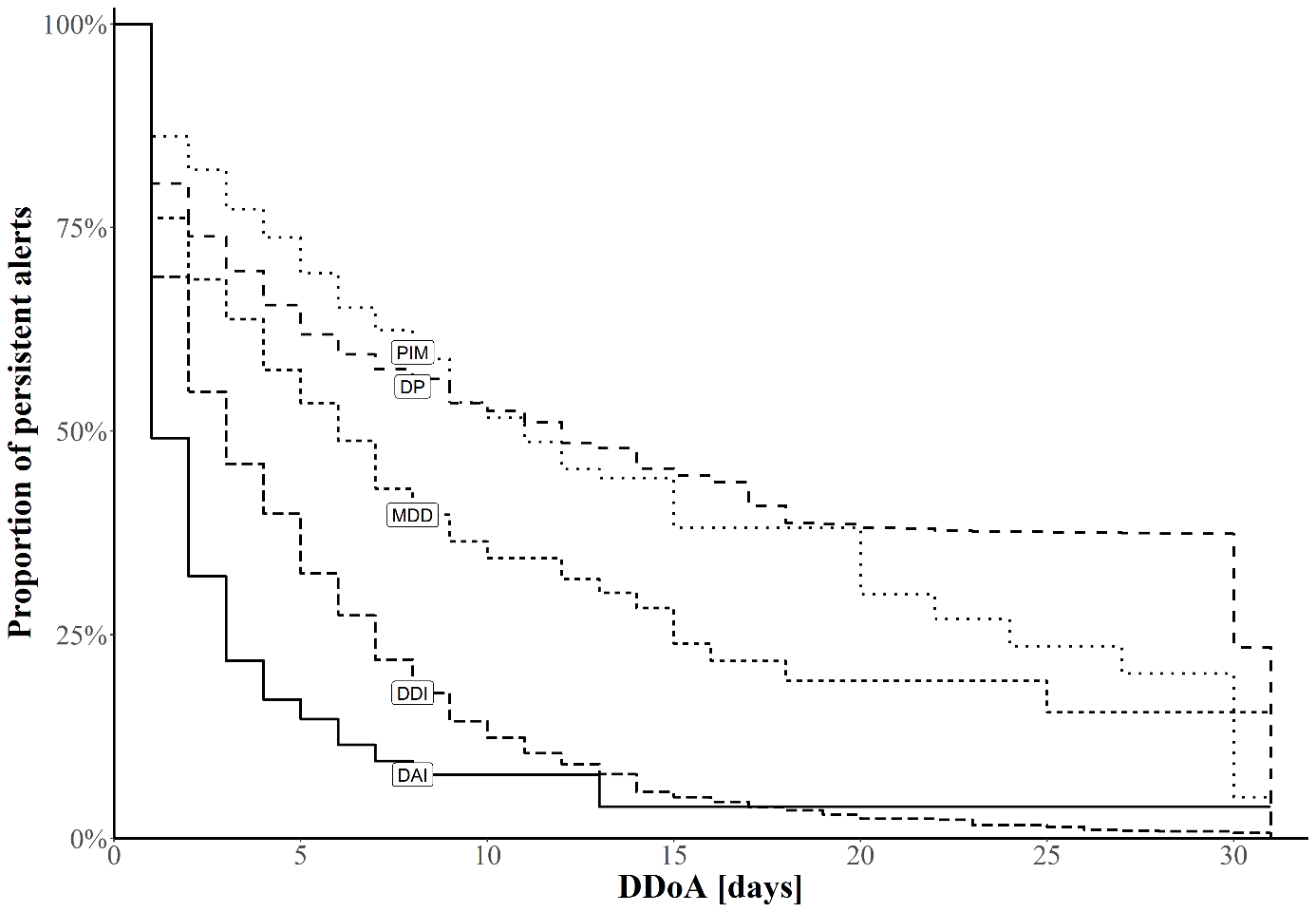


***Figure S5: Proportion of persistent alerts over the displayed period stratified by the alert type.*** *In this Kaplan-Meier chart it is assumed that 100 % corresponds to all alerts per alert type (i.e., 2,366 alerts for DPs or 499 for PIM), events correspond to absent alerts, and censoring corresponds to persistent alerts at the end of the observation period. Thus, each step visualizes the proportion of persistent alerts over the display duration of alerts (DDoA). The median persistence of each alert type varies in ascending order: DAI (1 day), DDI (3 days), MDD (6 days), PIM (11 days), and DP (12 days).*

***Abbreviations:*** *DAI: Alerts for drug-allergy interactions, DDI: Alerts for drug-drug interactions, DDoA: Display duration of alerts, DP: Alerts for duplicate prescriptions, MDD: Alerts for prescriptions exceeding the maximum recommended daily dose, PIM: Alerts for potentially inappropriate medication for patients aged ≥ 65 years*
